# Online Intelligent Controllers for an Enzyme Recovery Plant: Design Methodology and Performance

**DOI:** 10.4061/2010/250843

**Published:** 2010-12-27

**Authors:** M. S. Leite, T. L. Fujiki, F. V. Silva, A. M. F. Fileti

**Affiliations:** School of Chemical Engineering, Department of Chemical Systems Engineering, University of Campinas (UNICAMP), 13083-970 Campinas, SP, Brazil

## Abstract

This paper focuses on the development of intelligent controllers for use in a process of enzyme recovery from pineapple rind. The proteolytic enzyme bromelain (EC 3.4.22.4) is precipitated with alcohol at low temperature in a fed-batch jacketed tank. Temperature control is crucial to avoid irreversible protein denaturation. Fuzzy or neural controllers offer a way of implementing solutions that cover dynamic and nonlinear processes. The design methodology and a comparative study on the performance of fuzzy-PI, neurofuzzy, and neural network intelligent controllers are presented. To tune the fuzzy PI Mamdani controller, various universes of discourse, rule bases, and membership function support sets were tested. A neurofuzzy inference system (ANFIS), based on Takagi-Sugeno rules, and a model predictive controller, based on neural modeling, were developed and tested as well. Using a Fieldbus network architecture, a coolant variable speed pump was driven by the controllers. The experimental results show the effectiveness of fuzzy controllers in comparison to the neural predictive control. The fuzzy PI controller exhibited a reduced error parameter (ITAE), lower power consumption, and better recovery of enzyme activity.

## 1. Introduction

The present study is concerned with the design and experimental testing of intelligent control systems for temperature control in the precipitation plant of bromelain enzyme recovery. This biotechnological process may be considered the first step in the downstream processing of the protein. It is motivated by the high commercial value of this enzyme, the increasing demand for bromelain in pharmaceutical and industrial applications [1, 2], and the fact that bromelain can be recovered from kitchen waste (pineapple stem and rind).

The aim of the precipitation process is to achieve separation of solutes by conversion to solids. Precipitants can be chosen which do not denature the biological product, and the precipitate is often more stable than the dissolved form. Although precipitation is a simple operation, in the recovery of bromelain from pineapple, temperature control is crucial to avoid irreversible protein denaturation and hence improve the precipitation yield and the enzyme activity of the product [3]. 

Despite that automation and process control can significantly influence the yield and final quality of bioproducts, there are few experimental studies on the application of automatic controllers in the bioprocesses. Most works focus on results obtained from computational simulations, which indeed do not represent these processes in all their complexity. The transient behavior and nonlinearities of these processes make the design of classical control dependent on trial-and-error methodology, showing limited performance. On the other hand, intelligent controllers based on fuzzy logic and neural networks can be applied successfully to both linear and nonlinear systems. The main advantage of intelligent controllers is that modeling, based on first principles, is not required. Implementation of these controllers in small-scale pilot plants is essential to evaluate their potential value.

The wide range of existing fuzzy control applications indicates that this technique is gaining considerable importance in the control of complex processes and represents a promising approach to solving industrial control problems [4–9]. Some successful fuzzy logic experimental applications in biotechnological processes are reported in the literature by Steyer et al. [10], Martínez et al. [11], Sousa and Almeida [12], Babuska et al. [13], Horiuchi and Kishimoto [14], Traoré et al. [15], and Fileti et al. [16]. The computational simulation of a semibatch reactor stationed at a pharmaceutical company was used by Dovžan and Škrjanc [17] for testing a predictive functional control based on an adaptive fuzzy model. The goal was to control the temperature of the ingredients stirred in the reactor's core, so that they synthesize optimally into the final product. In order to achieve this, the temperature had to follow, as accurately as possible, the prescribed reference trajectory, with as low overshoot as possible.

Castañeda-Miranda et al. [18] developed a greenhouse intelligent climate control system that uses a fuzzy controller, based on a field programmable with a great potential for use in agricultural technology development due to its characteristics to produce fast prototypes of complex hardware designs with an effective production cost.

Lopes et al. [19] implemented an adaptive neurofuzzy inference mechanism (ANFIS) in a system of coupled tanks to simulate a production unit that used the level of the tanks for production and concentration control (process specifications). This inference system was proved efficient in aiding and replacing human workers for establishing relationships between process specifications and controlled variables (levels). Pires and Nascimento Júnior [20] simulated a control system of a robotic arm using a neurofuzzy system as a feedforward controller. The proposed control scheme was suitable to the robotic arm adjustment, obtaining a trajectory very close to the reference.

Hussain [21] selected in his review twenty four works concerning online application of neural networks. Many of them showed a better performance of the neural controller over the conventional one. Other studies proved the effectiveness of neural networks in modeling nonlinear processes, such as fermentation and polymerization processes [22, 23].

In this context, this paper describes the design methodology and a comparative study on the performance of fuzzy, neurofuzzy, and neural network intelligent controllers. They were implemented in order to maintain the temperature of the bromelain precipitation process from aqueous extract of pineapple wastes. The digital control was carried out by means of a Foundation Fieldbus communication system. To assess the performance of the digital controllers, the following parameters were used: overshoot, ITAE (integral of Time multiplied by Absolute Error), response time, enzymatic activity of the product and electric power consumption of the cooling system. The novelty of this paper is that, currently, there are no experimental studies about automation and process control in the production of bromelain, despite the growing number of scientific papers related to this enzyme.

## 2. Material and Methods

### 2.1. Precipitation Plant Description

**Figure 1:**
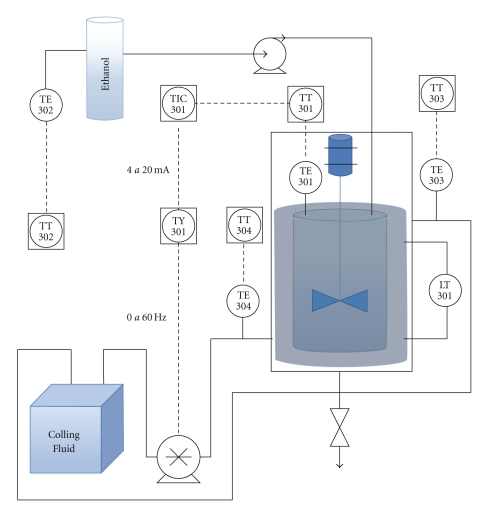
Fed-batch precipitation system flowchart.

Digital controllers were developed, implemented in the computer, and experimentally tested in a pilot plant of the precipitation process, outlined in Figure 1, located in the *Automation and Process Control Laboratory* at the *School of Chemical Engineering*, at the *University of Campinas* (UNICAMP).

**Figure 2:**
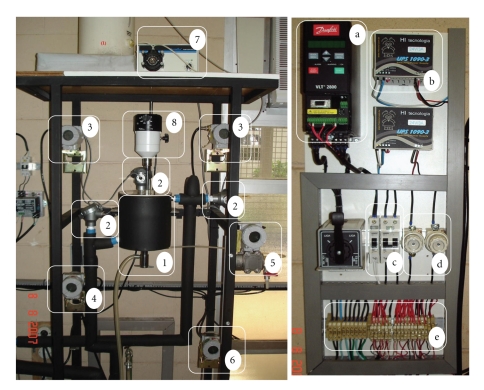
Pictures of (a) bromelain precipitation plant and (b) electrical control panel.

In Figure 2, it could be seen that the experimental system consisted of a stainless steel stirred tank with nominal capacity of 1000 mL (no. 1); a storage tank of the precipitating agent (ethanol 99.5 GL); a variable speed pump (1000 L/h maximum capacity), to enable the flow of the cooling fluid (50% v/v water/propylene glycol solution) through the tank jacket; a micropump that continuously fed the ethanol into the precipitation tank; four Pt-100 resistance thermometer detectors (no. 2)—TE 301, TE 302, TE 303, and TE 304 represented in Figure 1—to monitor the temperature in the precipitation tank, ethanol storage, the coolant outlet and inlet, respectively; two temperature transmitters (TT 302 device, Smar) (no. 3), with digital output signal, connected to the Pt100; a Fieldbus-current converter (no. 4) (FI302 device, Smar), coupled to a frequency converter; a level transmitter (no. 5)—LT 301, in Figure 1—which consists of a differential pressure transmitter (LD 302 device, Smar); a current-Fieldbus converter (no. 6) (IF302 device, Smar) to transmit signal of the power consumed by the coolant pump; a peristaltic pump (Masterflex Pump) for continuous feeding of ethanol in the precipitation tank (no. 7); a mechanical stirrer (no. 8). An electrical control panel (Figure 2(b)) was built, where it can be observed (a) the frequency converter (Danfoss VLT 2800), that drives the coolant pump speed (manipulated variable) (b) an electrical source for powering the field devices, (c) circuit breakers, and (d) fuses and (e) connectors, to allow corrective maintenance of equipments.

### 2.2. Operating Conditions of the Pilot Plant

The stem and rind of “Pérola” pineapples were ground and mixed to a uniform suspension in distilled water, at a dilution rate of 1 : 1 v/v. Solids were filtered from the mixture through a 0.45 **μ**m paper filter. The filtrate, called pineapple extract, contained the bromelain enzyme. Aliquots of 150 mL of pineapple extract were frozen at −18°C [24] until they were defrosted and individually used in the experiments.

Samples of 150 mL of pineapple aqueous extract were fed into the tank. A micropump was employed to feed the ethanol into the tank continuously at room temperature (approximately 23°C), at a fixed rate of 2.16 L/h, until the liquid volume reached 750 mL, corresponding to a 1 : 4 v/v ratio between extract and ethanol—the optimal condition for protein precipitation [24]. In order to avoid protein denaturation, the coolant flow rate was manipulated by means of a variable-speed pump, to maintain the temperature constant into the tank.

A design of experiments was proposed to find the nominal conditions for the following process variables: the ethanol micropump flow rate, the coolant pump flow rate (manipulated variable), and the coolant inlet temperature. Those nominal conditions were determined by maximizing the temperature deviation from set point and minimizing the rise time of temperature, which turns the process a challenging problem of control. The best values found were, respectively, 2.16 L/h, 374 L/h, and 0°C. The stirring rate was kept constant (150 rpm) during tests. The batch operation took about 17 minutes (time of contact between solvent and proteins).

### 2.3. Characterization of Bromelain

The temperature control has great influence on the final quality of the recovered bromelain enzyme (EC 3.4.22.4). Therefore, the determination of enzymatic activity was used as an important index of performance to compare the efficiency of the automatic controllers tested in this work.

The activity assay was based on the biuret colorimetric method. Under alkaline conditions, substances containing two or more peptide bonds form a purple complex with the copper salts in the reagent. The intensity of the color produced in the biuret reaction is proportional to the number of peptide bonds participating in the reaction. In this method, a unit (U) of enzyme activity is defined as the amount of enzyme able to change the absorbance reading at 540 nm by one unit in 10 min, at 37°C. The specific enzymatic activity, AE (U/g), is the relationship between the number of units of enzyme activity (U/mL) and the amount of protein (g/mL) in the sample.

### 2.4. The Digital Control System

In order to provide communication between the process and the local area network, a Fieldbus interface was used. An OPC server was responsible for providing the control program (OPCclient) with current values of measured variables and for communicating the control action to the frequency converter. 

**Figure 3:**
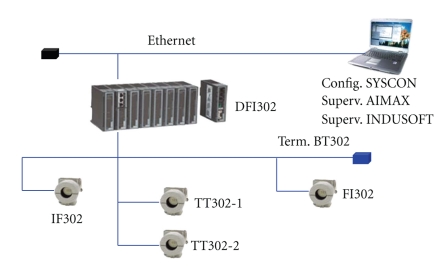
Fieldbus network for bromelain precipitation process.

The digital control was carried out through a Foundation Fieldbus communication system, as shown in Figure 3. 

The field devices composing the Fieldbus network, used to monitor and control the precipitation tank, were as follows

- Distributed Fieldbus interface (DFI302 device, Smar): it is manager of communication which controls the actions related to the Fieldbus system. It performs most functions required by the control system and connects the network of field devices to an Ethernet network.
- Current-Fieldbus converter (IF302): its allows the interconnection of instruments with analogue output 4 to 20 mA to a Foundation Fieldbus network. It has three independent channels.
- Fieldbus-current converter (FI302): it converts digital signals to analogue signals (4–20 mA). This device was connected to the frequency converter, taking information from the controller and changing the flow of coolant.
- Temperature transmitters (TT302): these devices have two channels that transform analog signals from temperature detectors to Fieldbus protocol.

The Software SYSCON 7.0 was used to configure Fieldbus network devices. This software allowed to make changes, maintenance, and operations on line. The main functional blocks of SYSCON software—resource, transducer, display, analog input, analog output, and signal characterizer—have been properly configured to achieve the desired functionality of each device. The software INDUSOFT Web Studio 6.1 was used for system management and data acquisition.

### 2.5. Controller Design

#### 2.5.1. Fuzzy PI Controller

This work was based on fuzzy logic concepts, wherein the knowledge accumulated by the process specialist was translated in a qualitative manner into a set of linguistic rules. To create a knowledge expert on the process, necessary for the development of fuzzy controller, experimental tests were performed to observe the behavior of the system.

In order to obtain a process reaction curve, a pseudosteady state condition was experimentally simulated by adding and withdrawing pineapple extract at the same rate (input and output) in the precipitation tank, without ethanol feeding. When the initial bulk temperature was close to the set point, ethanol began to be added at a fixed rate. At this exact moment, a disturbance of 30% of the maximum propylene-glycol pump capacity was applied. In this experiment, the pineapple extract was diluted with ethanol (1 : 1), in order to maintain the same precipitation conditions as during initial stages of the batch process. The system was monitored until the bulk temperature reached a new steady state.

The influence of the variation of the tank volume on the precipitation process was evaluated. Samples containing extract and ethanol in different proportions (from 1 : 1 to 1 : 3 v/v) were used in the pseudosteady state operation. Positive and negative disturbances were then applied (±30%) to the initial conditions of the speed of the coolant pump (manipulated variable). As in the fed-batch operation, a proportion of 1 : 1 corresponded to 300 mL, of 1 : 2 to 450 mL and of 1 : 3 to 600 mL. The process reaction curves obtained from this step contributed to the development of the Fuzzy controller.

**Figure 4:**
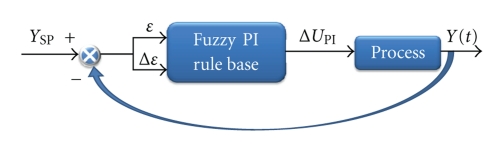
Structure of fuzzy PI control.

The error (*ε*) and the change of error (Δ*ε*) of bulk temperature, given by (1) and (2), respectively, were used as input linguistic variables. The speed variation of the coolant pump (Δ*U*), given by (3), was used as the output linguistic variable, resulting in a fuzzy PI incremental controller type. The structure of this controller is shown in Figure 4 and it was implemented using the Fuzzy Toolbox of the MATLAB 7.0.1 computer package.

The linguistic variables were defined by

(1) $$\begin{array}{c} \varepsilon \left(t\right)=Y\left(t\right)-Y_{\text{SP}}\left(t\right), \end{array}$$

(2) $$\begin{array}{c} \Delta \varepsilon \left(t\right)=\varepsilon \left(t\right)-\varepsilon \left(t-\Delta t\right), \end{array}$$

(3) $$\begin{array}{c} \Delta U\left(t\right)=U\left(t\right)-U\left(t-\Delta t\right). \end{array}$$

The rule base was composed of typical Mamdani-type rules for the inference engine in the fuzzy PI controller, employing the model proposed by Li and Gatland [25] and Li [26]. The original 49 rule proposals, from seven triangular membership functions (MF) for each variable, are presented in Table 1. The triangular membership functions were used in the fuzzification procedure because of their wide application in the literature and ease of implementation. This procedure comprises the process of transforming crisp values into grades of membership for linguistic terms of fuzzy sets. The linguistic expressions for the magnitudes of the linguistic variables contain the following seven adjectives: negative large (NL), negative medium (NM), negative small (NS), zero (ZR), positive small (PS), positive medium (PM), and positive large (PL).

Using the rule base of Table 1 and the triangular membership functions for the three linguistic variables, several rules are activated simultaneously, showing different membership degrees, or areas, for the output. In order to find a unique crisp value for the fuzzy PI output (Δ*U*), a defuzzification method was used based on the determination of the center of gravity of the combined areas:

(4) $$u^{*}=\frac{\sum_{i=1}^{N}u_{i}\mu_{\text{OUT}}\left(u_{i}\right)}{\sum_{i=1}^{N}\mu_{\text{OUT}}\left(u_{i}\right)},$$

where, *μ*_OUT_(*u*_*i*_) is the membership function area resulting from fuzzy PI inference operation min  (i.e., among the different areas obtained for Δ*U* calculation from simultaneous rules, the smallest one is chosen), *u*_*i*_ is the center of area of each output membership function area, and u* is the center of area below the combined output membership function areas found from the intersection operator application. Indeed, u* gives the controller action to be implemented in the process (Δ*U*).

#### 2.5.2. Neurofuzzy Controller

The database obtained from the application of Mamdani-type fuzzy PI structure (Section 2.5.1) was used for training, validation and tests of the neurofuzzy inference system (ANFIS). For the training set, the odd samples were used, for the validation the even samples, and for the tests, all the data. The ANFIS Toolbox of MATLAB software was used. Using the *anfisedit* command, the training and validation database were loaded, the Takagi-Sugeno fuzzy inference system (FIS) was created, and the training procedure was performed with 100 epochs. Following this training procedure, the ANFIS output was compared to the test outputs to check the controller performance. 

**Figure 5:**
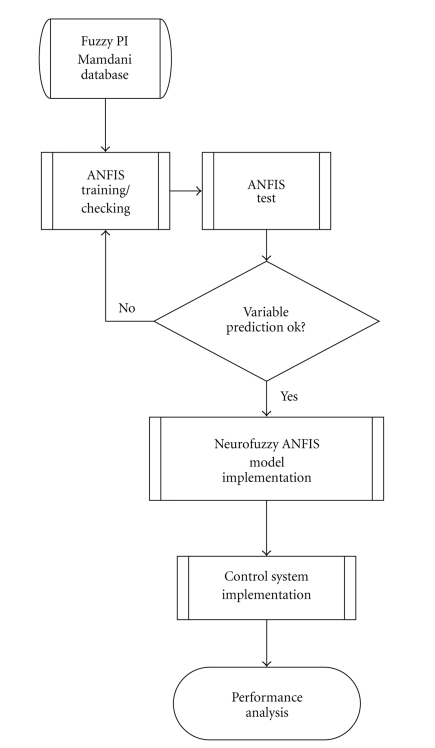
Flow chart for designing the neurofuzzy controller.

**Figure 6:**
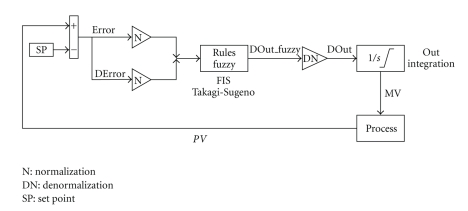
Neurofuzzy controller Simulink diagram, where Error, DError, and DOut are given by (1), (2), and (3), respectively, N and DN are fuzzyfication and defuzzification procedures (see Section 2.5.1), and SP is the set point of temperature.

The flowchart for designing the neurofuzzy controller is provided in Figure 5. Using Simulink/MATLAB and OLE for process control protocol (OPC), the controller was implemented online. The Simulink diagram of the neurofuzzy control system is provided in Figure 6.

#### 2.5.3. Model Predictive Controller (MPC) Based on Artificial Neural Networks (ANN)

Firstly, the process was modeled using the Neural Network Toolbox of MATLAB. In the input layer, the operating variables, measured every sample time *k*, were used. In the hidden layer, an activation function was applied to twice the number of nodes of the input layer. Another activation function was applied to the output layer, which predicts the one-step-ahead controlled variable (PV_*k*+1_). 

Open-loop runs were used to train the multilayered feedforward network, employing the Levenberg-Marquardt algorithm (*trainbr* command in MATLAB). The open-loop data set was obtained by gathering a wide range of values of the input layer variables, including the whole network action domain. 

**Figure 7:**
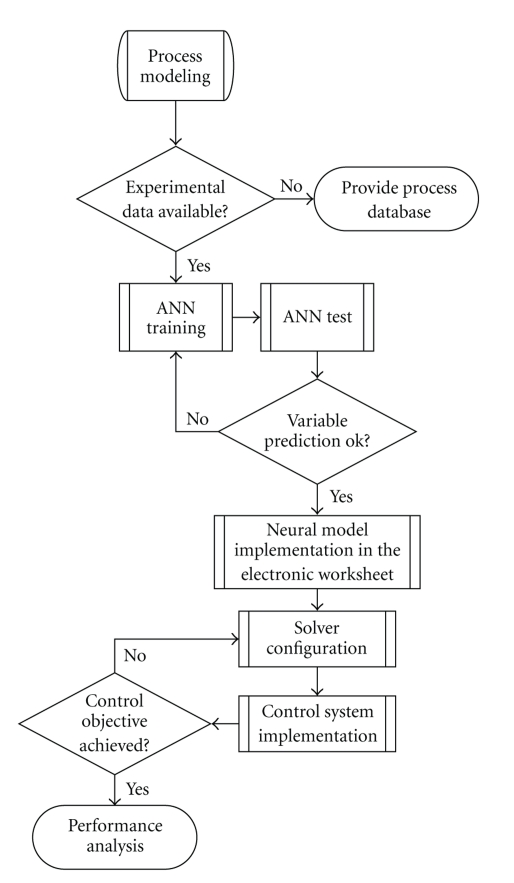
Flowchart for designing the MPC.

The process dynamic was initially observed from the open-loop run with the manipulated variable at a fixed point. From there, the step disturbances in the manipulated variable, *U*, were planned so that the controlled variable behavior could be monitored from several runs and this database was employed to train the neural network. The database was split in two sets: 75% and 25% for training and tests, respectively. Furthermore, closed-loop runs with a fuzzy PI controller were used for the tests as well, since the neural model was expected to have good response in closed-loop situations with the implementation of the MPC optimizer for the process control. The neural model performance was assessed through dispersion plots of the testing runs (network output *versus* target vector), with a suitable result being proved by a slope coefficient of the linear fitting of the dispersion plots close to the unity and the linear coefficient around zero. The flow chart for designing this alternative control system is provided in Figure 7.

**Figure 8:**
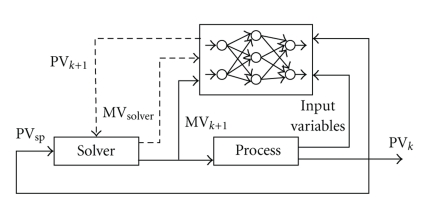
MPC diagram based on ANN predictions (one-step-ahead model predictive control), where MV is the manipulated variable *U*.

The optimized weights and biases of the trained network were inserted in an electronic worksheet to reproduce the algebraic equations of a neural model. The calculated output generates a quadratic error (relative to the set point), which was defined as the objective function to be minimized by the solver (5). The solution was found from the quasi-Newton method of generalized reduced gradient, available in Excel software, by changing the manipulated variable value that should be implemented in the plant (*U*). The MPC diagram of the overall control system is provided in Figure 8:

(5) $$\underset{U}{\min\;}{\left({\text{PV}}_{\left(k+1\right)}-{\text{PV}}_{\text{sp}}\right)}^{2}$$

subject to the following constraints:

(6a) $$\begin{array}{c} U\geq U_{\min\;}, \end{array}$$

(6b) $$\begin{array}{c} U\leq U_{\max\;}, \end{array}$$

(6c) $$\begin{array}{c} \left|U_{\text{solver}}-U_{k-1}\right|\leq \;\text{maximum}\;\text{step}\;\text{allowed}. \end{array}$$

## 3. Results and Discussion

### 3.1. Determination of Set Point

**Figure 9:**
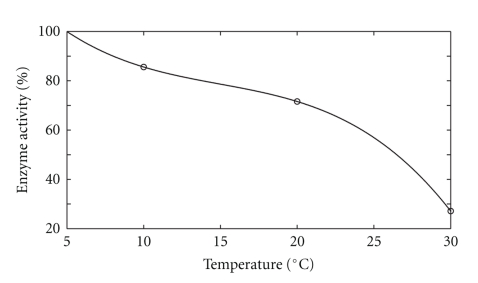
Temperature influence over bromelain enzyme activity.

To avoid denaturation of the protein, the temperature set point was determined from the literature and also from experimental testing of enzymatic activity to examine the quality of the enzyme. The results showed an activity of 0.8739 U/mL at 5°C, 0.7478 U/mL at 10°C and 0.7204 at 20°C (Figure 9), which shows that at lower temperatures the effect of denaturation is attenuated. Those results were very close to those obtained by Cesar et al. [24], which do not recommend setting the temperature below 5°C. Thus, the set point selected for the precipitation temperature was 5°C.

### 3.2. Controller Design

#### 3.2.1. Fuzzy PI Controller

**Figure 10:**
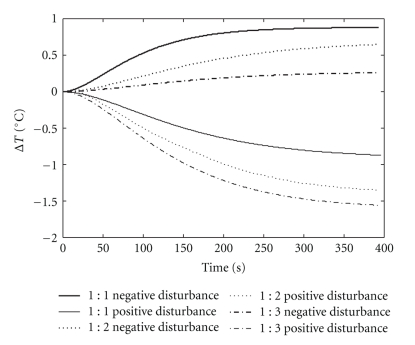
Reaction curves obtained from disturbances in the manipulated variable.

According to the procedure described in Section 2.5.1, the reaction curves were obtained and are shown in Figure 10. The initial conditions of the tests were kept in 40% of rotation of the pump and 5^°^C for the temperature inside the tank. 

By observing the reaction curves (Figure 10), it was clear that the absolute value of the process gain (*K*_*p*_) increased as tank volume rose up, for positive disturbances (high speed of coolant pump). On the other hand, the absolute gain decreased as the volume increased, for negative disturbances (low speed). Thus, the temperature responses to positive and negative disturbances, of the same intensity, were asymmetrical.  Table 2 summarizes the values of static gain from the Figure 10. 

Two main factors determine these transient and nonlinear features of the process.

- Thermal exchange: at the beginning of the fed-batch process the heat exchange is deficient, as a consequence of the small heat transfer area. As the volume rises up, this area grows and heat exchange becomes more efficient. In the final condition of volume of 600 mL (1 : 3 curve), when applying a positive disturbance (high speed), there was a greater decrease in temperature inside the tank.
- Heat of dissolution of the alcohol: an effect that increases the nonlinearity of the process is the heat of dissolution of ethanol in aqueous solution. The amount of heat released is greater at the beginning of the process and this effect decreases continuously during precipitation. In the condition of volume of 600 mL (1 : 3 curve), the increase in temperature caused by the dissolution of ethanol in the solution was negligible.

Due to the transient behavior and nonlinearities, discussed above, the precipitation system is characterized by having different sensitivities to the control actions, which emphasizes the limitation of the use of conventional controllers in this system. To minimize the consequences, the procedure of tuning the fuzzy PI controller involved changes in the universe of discourse, rule base, and support sets of membership functions, based on the reaction curves analysis.

The original rules of Mamdani, shown in Table 1, were modified based on tests performed experimentally to provide better tuning of fuzzy PI controller. Adjustments are in bold in Table 3 and explained in the text above.

When the error was large positive (PL)—temperature was above the set point—and the change of error was NL, NM, or NS—the temperature was decreasing—there was not a need for great action in the coolant pump, as suggested by the original rules (Table 1). The heat exchange was becoming more efficient due to the enlargement of the volume and also due to the lower impact of dissolution of ethanol on the solution. Thus, these three actions were modified to NS, smoothing the control action that reached upper limit without saturation.

When the temperature was slightly above the set point (error PS) and still increasing Δ*ε* = PL, there was a need for positive action on the pump speed. However, as the error was still small, it was observed that a medium positive action (PM) met the same goal as large action (PL), reducing temperature oscillation around set point.

When the temperature was slightly below the set point (error NS), and increasing rapidly (Δ*ε* = PL), it was observed that a small positive (PS) increase in pump speed would be enough to stop this temperature increase, since the gain of the process was large at the final conditions of batch. The rule medium positive (PM) originally suggested in Table 1 could provide a significant reduction in temperature and might cause undesirable oscillations. For the same reasons as outlined above, when error was negative medium (NM) and change of error was large positive (PL), the rule was changed to ZR, because maintaining the coolant flow rate showed to be sufficient to prevent any rise in temperature.

**Figure 11:**
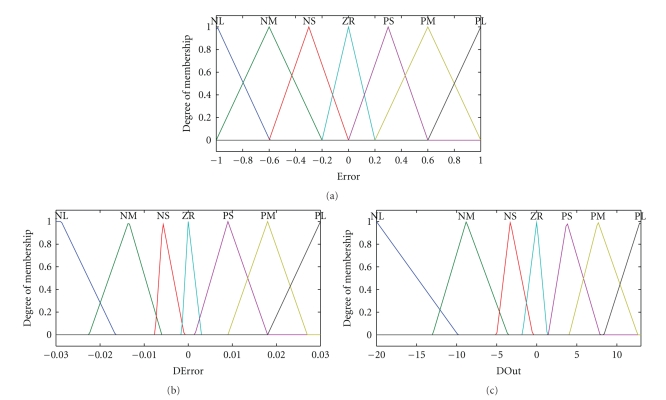
Membership functions of *ε*, Δ*ε*, and Δ*U*.

The triangular membership functions, and their corresponding labels of error (*ε*), change of error (Δ*ε*), and the incremental control action (Δ*U*), were presented in Figure 11. 

It may be noted in Figure 11 that the universe of discourse of error has a narrow range (from −1 to 1), since it was observed that the beginning of the process was the most critical stage, demanding a fast response of the manipulated variable, even for small errors (*ε*) in the controlled variable. Thus, even for small variations in the controlled variable, an intense response of the manipulated variable is produced.

Based on the analysis of the dynamic behavior of the process, a wide range (from −20 to 13) was set for the output variable (Δ*U*). In terms of absolute magnitudes, the lower bound (−20) was greater than the upper bound (13), owing to the need to reduce the pump speed more quickly when the process reaches maximum error. In this region, lower coolant flow rates were able to handle the temperature rise caused by the release of heat of dissolution of the alcohol during the precipitation process.

**Figure 12:**
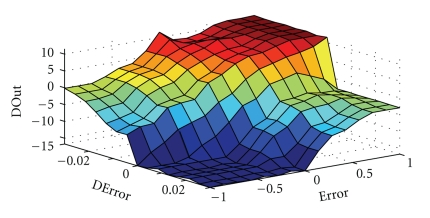
Fuzzy PI controller control surface.

The control surface related to the fuzzy PI controller is represented in Figure 12, where nonlinearities incorporated in this control system are clearly observed.

#### 3.2.2. Neurofuzzy Controller

The ANFIS controller design consisted in finding a Takagi-Sugeno (TS) fuzzy structure, which was able to describe an incremental fuzzy PI controller action, by choosing as linguistic variables *ε*, Δ*ε*, and Δ*U* ((1), (2), and (3)). The FIS file was made up of three triangular membership functions (ZR: approximately zero, PS: positive small, and PL: positive large) for the error (*ε*), seven triangular membership functions (NL: negative large, NM: negative medium, NS: negative small, ZR: approximately zero, PS: positive small, PM: positive medium, and PL: positive large) for the change of error (Δ*ε*), and twenty-one linear functions for the pump speed variation (Δ*U*). Another FIS file was created, for comparison of performances, with four triangular membership functions for the error (*ε*) (including PM: positive medium). The universes of discourses were determined according to subjective knowledge of the process and they were similar to that used in the fuzzy PI Mamdani controller (Figure 11). 

**Figure 13:**
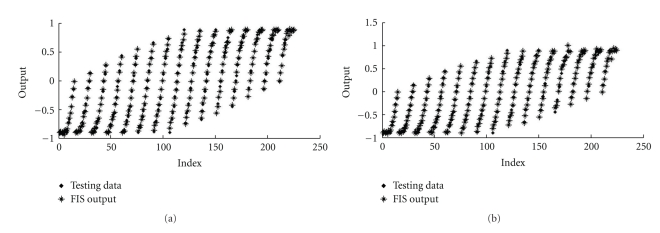
Testing data from FIS TS output *versus* test samples of FIS output, using (a) three membership functions (ANFIS_3MF_) and (b) four membership functions (ANFIS_4MF_).

After the training procedure, the ANFIS controller outputs (FIS TS output) were tested with the samples obtained from the fuzzy PI Mamdani structure (FIS output), which allowed the observation of the high training performance. In Figure 13, the FIS TS outputs are plotted *versus* the test samples (index). It could be noted that the points are coincident, even for the validation set that uses unseen data.

#### 3.2.3. Model Predictive Controller

The operating variables for the ANN input layer, measured every four seconds (sample time), were chosen as follows: ethanol temperature (*T*_alc,*k*_), which exerts great influence in the precipitation temperature—the higher the ethanol temperature, the higher the overshoot obtained; coolant inlet (*T*_in,*k*_) and outlet (*T*_out,*k*_) temperatures, which provide information on the heat exchange in the tank jacket; coolant pump speed (*U*_*k*_), that is, the manipulated variable, so that its value will determine the controlled variable response; the liquid level (*L*_*k*_), represented by the liquid volume, provides the ANN with information on the run time, thus distinguishing equal input vectors that correspond to different output vectors; pump speed variation (Δ*U*_*k*_) that indicates to the ANN which step disturbance in pump speed caused the given output; bulk temperature (*T*_bulk,*k*_), which works as a reference for the ANN prediction of the one-step-ahead bulk temperature (*T*_bulk,*k*+1_), that was chosen as the output variable of the ANN. In the hidden layer a hyperbolic tangent activation function was applied to fourteen nodes. 

**Figure 14:**
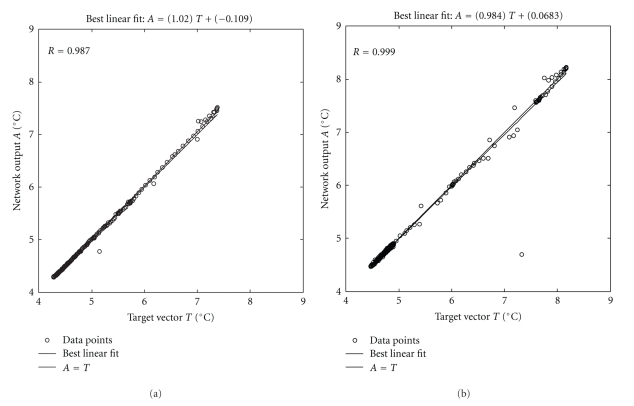
Dispersion plot of the network prediction output *versus* the target vector for (a) an open-loop run and (b) a closed-loop run.

Figure 14 shows the results for the open-loop and the closed-loop offline tests of the neural model. These tests, with unseen data, proved that the ANN successfully predicted the tank temperature, as it can be seen by the agreement between actual (target) and predicted values of temperature. Both linear fits presented slope coefficients close to the unity and linear coefficients around zero, approaching the diagonal line. 

The optimized weights and biases of the trained network were then inserted in an electronic worksheet to reproduce the algebraic equations of the neural model. Simultaneously to the experiment, the neural model was able to predict on line the one-step-ahead bulk temperature. The results showed that the ANN was capable of learning the inherent nonlinearities and also successfully predicted the bulk temperature, thus being considered suitable for the MPC application. 

### 3.3. Closed-loop Experimental Tests

The inlet ethanol flow rate at room temperature, the volume, and the heat transfer area variations were inherent disturbances, which drove the process away from the set point. All experimental tests were carried out under the same experimental conditions. 

**Figure 15:**
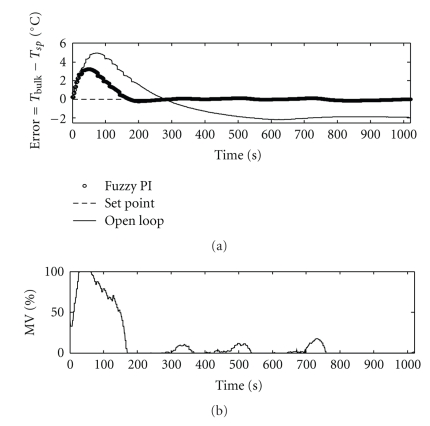
Behavior of the controlled and manipulated variables under fuzzy PI Mamdani control.

Figure 15 shows the temperature deviation under a well-tuned fuzzy PI Mamdani controller. Final settings were presented in Figure 11 and Table 3.

**Figure 16:**
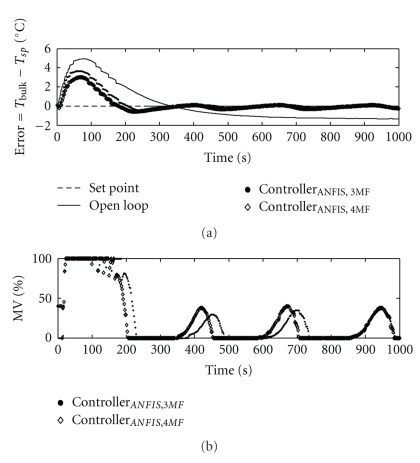
Behavior of the controlled and manipulated variables under neurofuzzy control.

Since the neurofuzzy tests showed the effectiveness of the training (Figure 13), the ANFIS controllers were applied in the plant. The results obtained are shown in Figure 16.

In Figure 16, it can be noted a suitable performance from both ANFIS controllers implementation, with three and four membership functions. By adding one membership function, the overshoot and the rise time were shortened.

**Figure 17:**
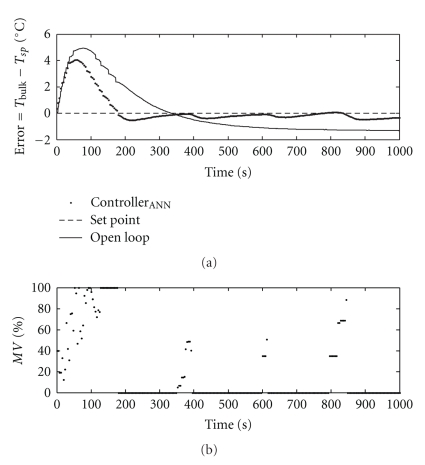
Behavior of the controlled and manipulated variables under MPC.

The neural model coupled with the Microsoft Excel solver was used as a MPC temperature controller, and the experimental results are shown in Figure 17. The manipulated variable, to be changed by the solver, was subject to the following constraints (5): range of 0 (*U*_min_) to 100% (*U*_max_) of speed variation; manipulated variable action was smoothed by restricting its step value up to 35%. To prevent the controlled variable from leaving the training operating range, an additional constraint was added: the pump was turned off when the bulk temperature reached 4.9°C.

Figure 17 shows that the developed MPC controller was able to maintain the controlled variable around set point (5°C), with small rise time. As discussed in Section 3.2, the overshoot observed in the first 200 seconds is due to the low liquid level in the tank, which caused the heat exchange area to be at a minimum. The dissolution heat produced during the ethanol addition can explain it as well.

Table 4 summarizes some important indexes in order to compare the performance of the implemented controllers. The best controller, in terms of overshoot and rise time, was the fuzzy PI Mamdani one.

The early stage of ethanol addition is critical. In order to keep the overshoot to a minimum, intense controller response is required, causing pump saturation, which was noted under all control strategies (Table 4). However, the saturation effect was far less noticeable when the fuzzy PI controller was used, favoring conservation of the equipment.

 Some other quantitative and qualitative analysis on the performance of the best implemented controller is summarized in Table 5. It could be observed very low power consumption under fuzzy PI control.

The enzymatic activity analysis proved that fuzzy PI control outperformed the other implemented controllers because the final product (bromelain enzyme) showed high activity. From Figure 15, it was observed the stable maintenance of bulk temperature, resulting in high quality of the product. 

## 4. Conclusions

We have described the design and experimental testing of intelligent control algorithms for temperature control during the precipitation of bromelain with solvent. The temperature was monitored and controlled in order to minimize bromelain denaturation during the precipitation process. 

Tuning the controllers proved to be a difficult task in this fed-batch nonlinear process. Fuzzy tuning was hindered by the simultaneous multiple adjustments. Nevertheless, the procedure based on the analysis of the process reaction curves proved to be an attractive strategy to provide a suitable nonlinear controller design for transient processes.

From the results, it was concluded that all proposed controllers were suitable for the precipitation tank temperature control. The fuzzy PI Mamdani controller showed better global performance criteria: small ITAE, short response time and pump saturation time, and higher enzyme activity in the product. This fuzzy PI controller also presented lower power consumption, providing a significant reduction of operating costs. This high performance of the fuzzy PI controller can be attributed to its ability to adapt to the nonlinearities.

The real-time data exchange between the softwares MATLAB and Indusoft and also between the softwares and the field devices showed to be reliable and fast through the implemented Fieldbus architecture. 

The developed controllers gathered the benefits of the artificial intelligence in affording nonlinearities, making this methodology a promising new way to face complex process control problems, without spending efforts unnecessarily in rigorous mathematical modeling.

## Figures and Tables

**Table 1:** Original rule base of the fuzzy PI controller.

Error (*ε*)	Change of error (Δ*ε*)						
	NL	NM	NS	ZR	PS	PM	PL
PL	ZR	PS	PM	PL	PL	PL	PL
PM	NS	ZR	PS	PM	PL	PL	PL
PS	NM	NS	ZR	PS	PM	PL	PL
ZR	NL	NM	NS	ZR	PS	PM	PL
PS	NL	NL	NM	NS	ZR	PS	PM
NM	NL	NL	NL	NM	NS	ZR	PS
NL	NL	NL	NL	NL	NM	NS	ZR

**Table 2:** Static gains of the process, *K*_*p*_, obtained from the reaction curves.

Disturbances		Positive	Negative

Volume (mL)		*K*_*p*_ (^°^C/%)	*K*_*p*_ (^°^C/%)
300	1 : 1	−0.030	0.030
450	1 : 2	−0.047	0.020
600	1 : 3	−0.053	0.010

**Table 3:** Modified rule base after tuning the fuzzy PI controller.

Error (*ε*)	Change-of-error (Δ*ε*)						
	NL	NM	NS	ZR	PS	PM	PL/%)
PL	**NS**	**NS**	**NS**	PL	PL	PL	PL
PM	NS	ZR	PS	PM	PL	PL	PL
PS	NM	NS	ZR	PS	PM	PL	**PM**
ZR	NL	NM	NS	ZR	PS	PM	PL
PS	NL	NL	NM	NS	ZR	PS	**PS**
NM	NL	NL	NL	NM	NS	ZR	**ZR**
NL	NL	NL	NL	NL	NM	NS	ZR

**Table 4:** Closed-loop performance parameters.

Controller	Overshoot (°C)	Rise time (s)	Pump saturation time interval (s)	Respective Figure
Fuzzy PI	3.1	170	35	15
ANFIS_3MF_	3.7	210	160	16
ANFIS_4MF_	3.0	180	140	16
MPC	4.1	180	135	17

**Table 5:** Complementary analysis for fuzzy PI controller.

Parameters	Open-loop	Fuzzy PI
Response time (s)	—	171
ITAE (×10^3^)	950.5	56.6
Specific enzymatic activity (U/g)	0.32	1.80
Electric energy consumption (kWh)	42	3.6

## Nomenclature

*K*_*p*_: Static process gain
*u*_ss_: Bias
*ε*: Temperature error
Δ*ε*: Change of error
Δ*U*(*t*): Change in control action
*U*(*t*): MV: control action
*Y*(*t*): PV: plant output
*μ*: *μ*_*i*_: Membership function (activation level)
ITAE: Integral of the time-weighted absolute error = ∫_0_^*∞*^ *t*||*e*(*t*)||*dt*
*Y*_sp_: Set point
*u**: Defuzzified action value
min: Minimum operator
OPC: OLE for process control
*i*: Index of discrete points
*N*: Negative membership function
*P*: Positive membership function.
